# Acute bilateral foot drop with or without cauda equina syndrome—a case series

**DOI:** 10.1007/s00701-021-04735-0

**Published:** 2021-02-07

**Authors:** Andreas K. Demetriades, Marco Mancuso-Marcello, Asfand Baig Mirza, Joseph Frantzias, David A. Bell, Richard Selway, Richard Gullan

**Affiliations:** 1 Department of Neurosurgery, New Royal Infirmary, Edinburgh, UK; 2 Department of Neurosurgery, King’s College Hospital, London, UK; 3 Department of Neurosurgery, Brighton and Sussex University Hospital, Brighton, UK

**Keywords:** Footdrop, Bilateral footdrop, Acute bilateral footdrop, Cauda equina syndrome, Lumbar stenosis, Lumbar disc prolapse, Lumbar disc prolapse, Degenerative spine disease, Surgical treatment, Timing of surgery

## Abstract

***Introduction*:**

Isolated acute bilateral foot drop due to degenerative spine disease is an extremely rare neurosurgical presentation, whilst the literature is rich with accounts of chronic bilateral foot drop occurring as a sequela of systemic illnesses. We present, to our knowledge, the largest case series of acute bilateral foot drop, with trauma and relevant systemic illness excluded.

***Methods*:**

Data from three different centres had been collected at the time of historic treatment, and records were subsequently reviewed retrospectively, documenting the clinical presentation, radiological level of compression, timing of surgery, and degree of neurological recovery.

***Results*:**

Seven patients are presented. The mean age at presentation was 52.1 years (range 41–66). All patients but one were male. All had a painful radiculopathic presentation. Relevant discopathy was observed from L2/3 to L5/S1, the commonest level being L3/4. Five were treated within 24 h of presentation, and two within 48 h. Three had concomitant cauda equina syndrome; of these, the first two made a full motor recovery, one by 6 weeks follow-up and the second on the same-day post-op evaluation. Overall, five out of seven cases had full resolution of their ankle dorsiflexion pareses. One patient with 1/5 power has not improved. Another with 1/5 weakness improved to normal on the one side and to 3/5 on the other.

***Conclusion*:**

When *bilateral* foot drop occurs *acutely*, we encourage the consideration of degenerative spinal disease. Relevant discopathy was observed from L2/3 to L5/S1; aberrant innervation may be at play. Cauda equina syndrome is not necessarily associated with acute bilateral foot drop. The prognosis seems to be pretty good with respect to recovery of the foot drop, especially if partial at presentation and if treated within 48 h.

## Introduction

Whilst the presence of slowly progressive bilateral foot drop is common in chronic systemic conditions, acute foot drop is a rare clinical presentation and acute *bilateral* foot drop is even rarer. An aetiology of degenerative spinal disease is rarer still. Only 6 cases have been reported in the previous literature [1, 12, 20, 21, 27].

The most common reports of *acute* bilateral foot drop are due to bilateral common peroneal nerve palsies [17–19, 22, 25, 36], notably due to iatrogenic compression during surgical positioning in a range of surgical specialties [2, 5–7, 10, 13, 16, 24, 32, 33].

We present 7 cases of *acute* and *bilateral* foot drop, all due to degenerative spinal disease, with trauma and relevant systemic illness excluded as causes. We aim to provide insight into the aetiology of acute bilateral foot drop from degenerative spinal causes and provide a schema with which to approach this rare, fairly obscure and challenging clinical presentation.

## Methods

Demographic and radiological data from seven cases of acute bilateral foot drop which presented to three neurosurgical centres over a 13-year period were prospectively collected by the treating surgical team. Complementary data was collected retrospectively. We reviewed patient demographics, clinical presentation, radiological parameters, and surgical parameters including time from presentation to surgery. Preoperative, early postoperative, and latest follow-up were compared.

## Results

The characteristics of each case in the series are shown in Table 1.

**Table 1:** Demographic, clinical, and radiological parameters at presentation, and subsequent follow-up

Patient	Age	Sex	Details of presentation	Disc level	Nature of disc prolapse	Pre-op dorsiflexion power (L, R)	Cauda equina syndrome	Procedure	Time from presentation to surgery (hours)	Post-op dorsiflexion power (L, R)	Latest follow-up (months)	Latest follow-up dorsi flexion power (L, R)	Impression at latest follow-up
1	57	M	● 8 day history of severe back pain and an electrifying sensation in the right leg ● worsening weakness in both legs for 3 days, worse on the right than on the left ● foot drop for 5 days at presentation ● bowel and bladder symptoms ● catheterized in hospital ● Saddle anaesthesia and a reduced anal sphincter tone	L2-3	Soft and central	(1/5,1/5)	Yes	L2-3 microdiscectomy and bilateral lateral recess decompressions via laminectomy	48	(1/5, 0/5)	6	(5/5, 5/5)	● No pain in the lower back or legs ● No bladder symptoms ● Difficulty with ejaculation whilst erection is unaffected ● Residual muscle weakness proximally in the hips, affecting his knee extension ● Perianal and perineal numbness ● Lower limb numbness ● Discharged from follow-up
2	42	M	● Reduced sensation and weakness of dorsiflexion in both feet ● Reduced mobility ● 3 weeks of worsening bilateral foot drop ● Intermittent paresthesia in right calf and left leg	L4-5	Soft and postero-lateral	(1/5,1/5)	No	Bilateral L4/5 microdiscectomy	24	(0/5, 0/5)	9	(5/5, 3/5)	● Significant improvement of pain ● Driving with a right ankle splint ● Discharged from follow-up
3	45	M	● Presented acutely with severe pain radiating to the lower limbs and bilateral foot drop ● 6-day history of right sciatica ● 3-day history of left sciatica ● Past surgical history included L5/S1 discectomy in 1990 due to low-grade lumbar pain and radiation to lower limbs	L3-4	Soft and postero-lateral	(1/5,1/5)	No	L3 laminectomy + L3/4 microdiscectomy and bilateral lateral recess decompressions	48	(1/5,1/5)	2	(5/5, 5/5)	● Independently mobilising ● Moving his feet independently with no foot drop ● Complains of residual pain and numbness affecting mainly the right leg ● Ongoing issues with mobility (due to numbness) and posture with ongoing physiotherapy ● Discharged from follow-up
4	41	M	● Presented acutely with low back pain ● Bilateral L5 radiculopathy ● Weakness in dorsiflexion slight worse on the right side ● No known previous medical conditions of note	L3-4	Soft and postero-lateral	(0-1/5, 0-1/5)	No	L3/4 microdiscectomy and bilateral lateral recess decompressions	24	(2/5,1/5)	113	(5/5, 5/5)	● No difficulty in walking ● Full return of power ● Reports complete resolution of symptoms as of 89 months post-op ● Discharged from follow-up
5	51	F	● Presented acutely with low back pain and bilateral foot drop ● No known previous medical conditions of note	L5-S1	Soft postero-lateral disc herniation + bilateral lateral recess stenosis	(3/5, 3/5)	Yes	L5/S1 microdiscectomy via L5 laminectomy and bilateral lateral recess decompressions	6	(5/5, 5/5)	2	(5/5, 5/5)	● No weakness ● No pain ● No sphincter problems ● Mild reduction in sensation on the lateral aspect of the right leg ● Discharged from follow-up
6	63	M	● Presented acutely with bilateral foot drop ● No known previous medical conditions of note	L3/4 disc and L3/4-L4/5 lateral recess stenosis	Soft postero-lateral disc herniation + bilateral lateral recess stenosis	(2/5, 2/5)	No	L3/4-L4/5 laminectomy and bilateral lateral recess decompressions and bilateral L3/4 microdiscectomy and foraminotomy	24	(4/5, 4/5)	12	(5/5, 5/5)	● No pain ● No weakness as of 3 months post-op ● Discharged from follow-up
7	66	M	● Severe acute low back pain radiating down both lower limbs, predominantly on the left ● Developed severe low back pain the day before with bilateral lower limb weakness and numbness on soles of the feet leading of a fall due to pain	L2-3 and L4/5 stenosis	Soft postero-lateral disc herniation + bilateral lateral recess stenosis	(1/5, 1/5)	Yes	L2/3 microdiscectomy and laminectomy and bilateral lateral recess decompressions and L4/5 laminectomy and and bilateral lateral recess decompressions	24	(0/5, 0/5)	2	(1/5, 1/5)	● Improvement in pain ● Mild improvement of ankle dorsiflexion ● Gluteal and hamstring muscle wasting ● No flickers of movement felt with active gluteal movement ● No sphincter improvement ● Follow-up ongoing

The mean age at presentation was 52.1 years (range 41–66). All patients but one were male. All seven had a radiculopathic presentation and three of these had concomitant cauda equina syndrome (CES).

Six of the seven cases had less than antigravity power at presentation: four patients with 1/5 power bilaterally; one with 0/5 power; one with 2/5. The seventh patient had 3/5 power.

All cases had painful foot drop. Two were operated within 48 h of presentation, whereas the remaining five were operated within 24 h.

Five out of seven cases had full resolution of their ankle dorsiflexion paresis. One patient with 1/5 power never improved. Another with 1/5 weakness improved to normal on the one side and to 3/5 on the other.

**Fig. 1:**
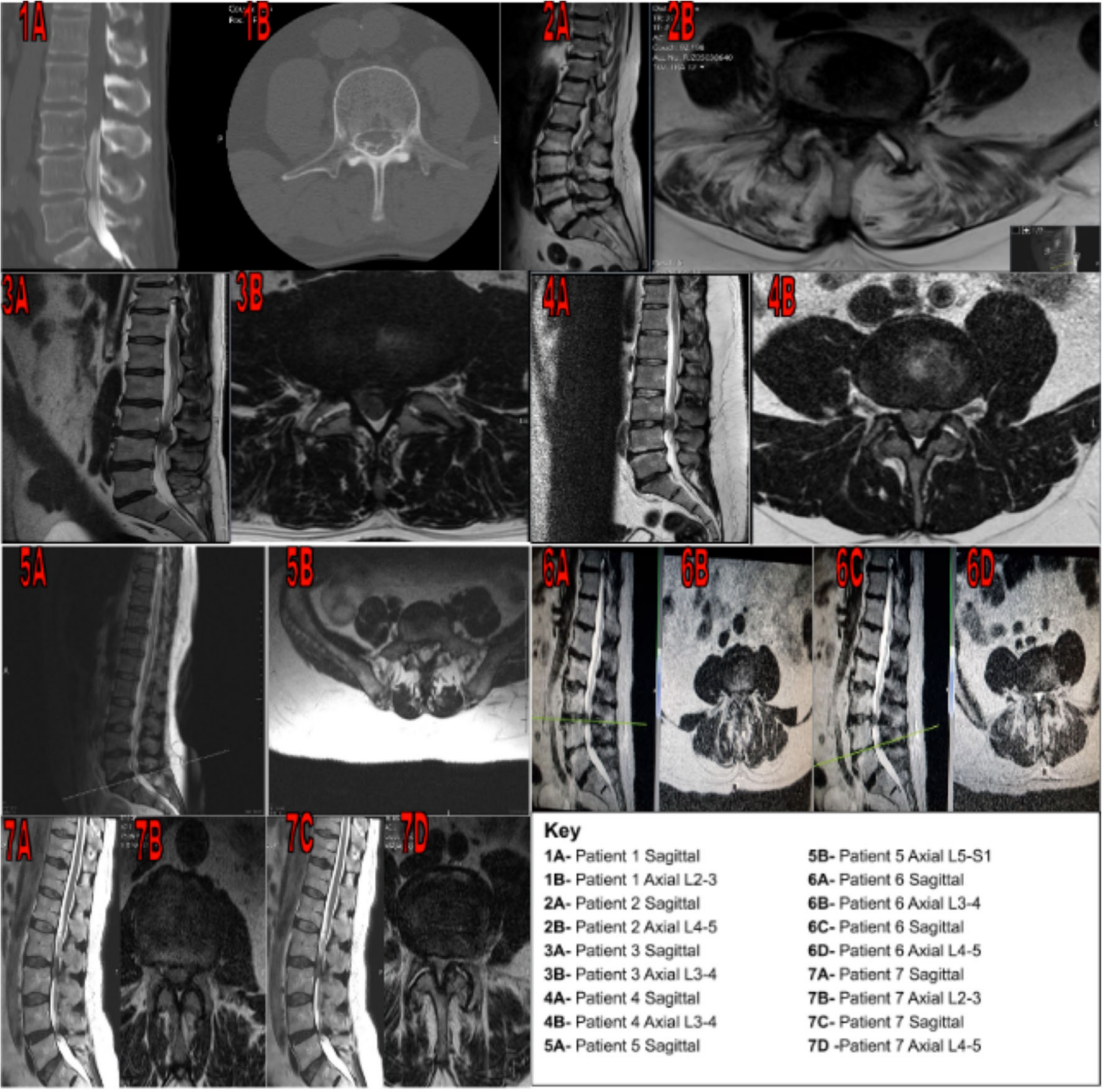
Sagittal and axial imaging views of the seven patients with acute bilateral foot drop. In patient 1, where an MRI was contra-indicated, a myelogram was performed. The levels affected ranged from L2/3 to L5/S1. The most commonly affected level was L3/4 in three out of the seven patients

Only three out of the seven cases had concomitant CES; the levels of compression in the first two cases were L2/3 and L5/S1, respectively, whilst the final patient had an acute disc prolapse at L2-3 with concurrent canal stenosis at L4/5. These are summarised in Fig. 1. The first two had full resolution of sphincter function, one by the 6 weeks follow-up and the other on the same-day post-op evaluation; both these have residual numbness; one of these had residual sexual dysfunction. The third and most recent patient has had minor improvements overall; however, the follow-up is short thus far and ongoing.

## Discussion

Foot drop is typically defined as significant weakness in ankle (+/- toe) dorsiflexion [39].

There is a variety of surgical and non-surgical differential diagnoses, and theoretically, any pathology affecting any part of the anatomical chain involved in dorsiflexion (brain, spinal cord, nerve roots, lumbosacral plexus, sciatic nerve, peroneal/fibular nerve, and the anterior tibialis muscle) may lead to foot drop.

There are many reports of *chronic* bilateral foot drop occurring as a sequela of medical illnesses. Endocrine causes include hypothyroid myositis [9] and diabetic peripheral neuropathy [31] whilst diseases which modulate nutritional intake such as Anorexia Nervosa [15] and Crohn’s disease [8] have been implicated too. Moreover, anterior horn cell disease such as in motor neuron disease [40], neuromuscular junction disease such as myasthenia gravis [11], peripherally demyelinating disease such as the Guillain-Barre syndrome [30], and myopathies such as muscular dystrophy [26] could feasibly present with a *gradual onset* bilateral foot drop.

Reports of traumatic brain injury, and an anterior communicating artery intracranial aneurysm, presenting with acute bilateral foot drop highlight the need to exclude cranial/central causes when no other pathology can be found [14, 28, 34]. Both the brain and spinal cord could be the source of the presentation due to specific vascular, neoplastic, infective, or demyelinating lesions.

The nerve root innervation supplying the tibialis anterior is predominantly L4 and L5, whilst some EMG studies have shown small amounts of nerve fibre recruitment from the L2, L3, S1, and S2 nerve roots [37]. The spectrum of cases in our series supports this pattern of nerve fibre recruitment. Our clinical expectation would be that, commonly, a postero-lateral disc protrusion at the L3/4 or L4/5 levels, or a far lateral disc protrusion at the L4/5 or the L5/S1 levels, could cause foot drop. However, acute far lateral disc prolapses are rare bilaterally.

Of the six bilateral foot drop cases due to degenerative disc disease in the literature, four were caused by bilateral postero-lateral disc prolapses at the L4/5 level [1, 12, 21, 27], one at the L3/4 level [20], and one at the T12/L1 level [12].

Of our seven cases, one was caused by an L2/3 disc prolapse, three at the L3/4 level (commonest), one at L4/5, and one at L5/S1 disc; one case had compression both at L2/3 and L4/5 levels. Therefore, the majority of both our cases and those reported in the literature align with expectation, but there certainly exist some unexpected disc level prolapses which may be accounted for by aberrant innervation.

Besides degenerative disc disease, other reported spinal aetiologies of acute bilateral foot drop include synovial cysts [3, 4] and an intradural haematoma [38]. An intradural tumour has been implicated in unilateral but not in bilateral foot drop [35].

Cauda equina syndrome is not necessarily associated with acute bilateral foot drop. It is interesting that only three out of the seven reported cases had concomitant cauda equina syndrome. This might be initially surprising, because anatomically a disc prolapse that is big enough to compress the foraminae bilaterally might be expected to protrude centrally too. However, there was no cauda equina syndrome in four out of seven cases; these had bilateral foraminal stenoses but no central disc prolapses. This is probably related to the presence of pre-existing narrow lateral recess anatomy, perhaps allowing an acute on chronic phenomenon.

Of note, five of the seven patients (two in primary care and three in the emergency department) needed to attend more than once before any onward referral for investigation was made. This may reflect some uncertainty amongst non-specialist colleagues on the frontline, which may be arising from a lack of clarity within the specialist (i.e., neurosurgical/spinal) community itself, where the timing of surgery for acute discogenic foot drop remains a point of discussion [23, 29]. We hope that the series presented will help in timely suspicion and investigation of acute lumbar spine aetiology.

**Fig. 2:**
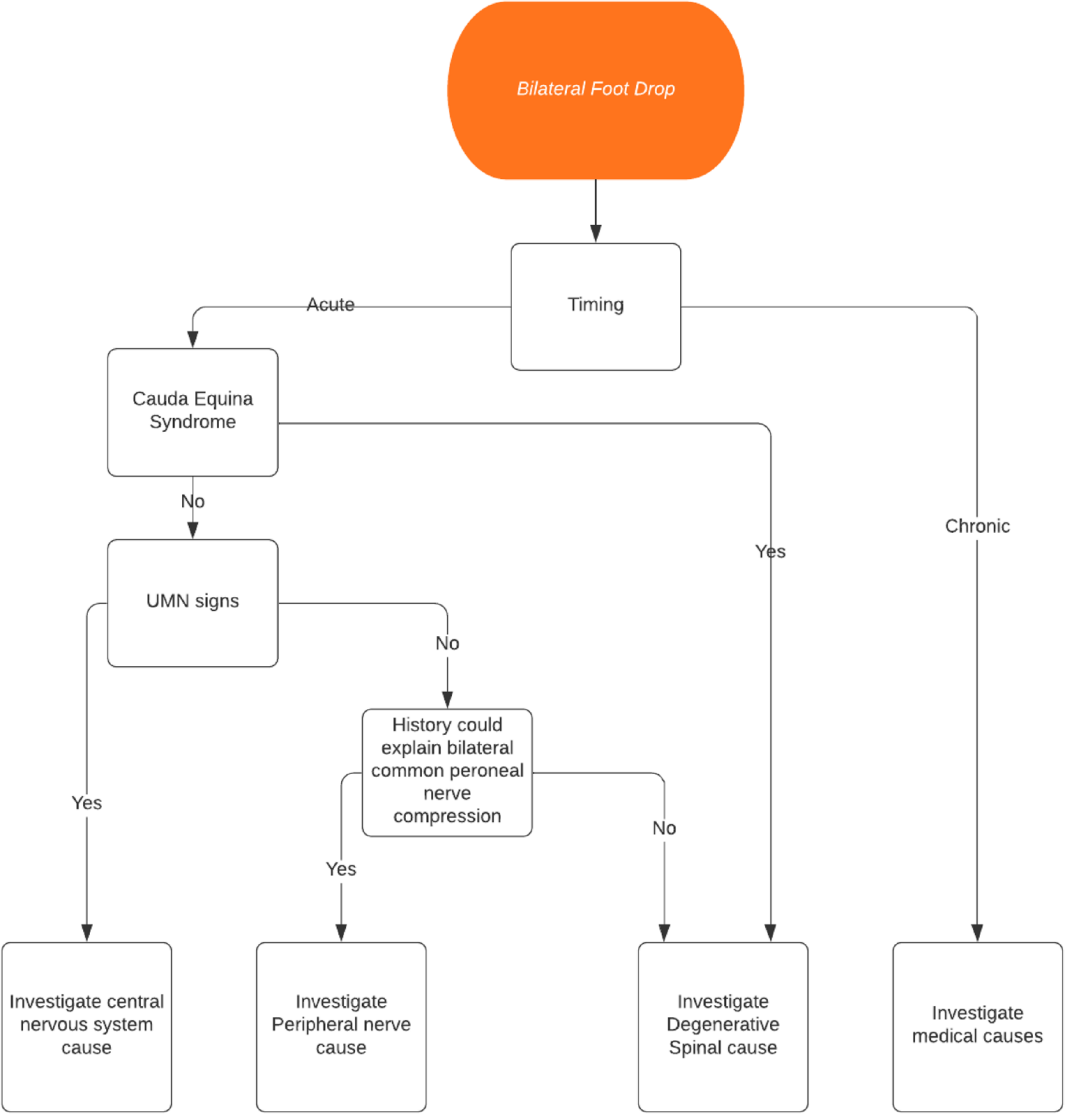
A flow chart illustrating a suggested work-up for bilateral acute foot drop presentation

A flow chart illustrating a suggested work-up for bilateral acute foot drop presentation is shown in Fig. 2. We believe that such patients ought to undergo surgical decompression of the nerve roots as soon as possible, to minimise the degree and duration of damage to the nerve, and hence improve the chance of recovery. However, neural recovery may be influenced by the presence of concomitant morbidity, including diabetes, obesity, and peripheral vascular disease.

### Limitations

The retrospective nature of this report is an obvious limitation. Could it be that the condition of acute and bilateral foot drop due to degenerative disc disease is not rare and may be underreported? One might consider that with unilateral foot drop being a much commoner situation, it may lower the interest of reporting bilateral cases. Or could it be that underreporting might be due to the fact that the line of conduct and recommendations for treatment do not differ? In our view, the reporting of seven cases encountered over 13 years suggests otherwise. Furthermore, the reality of the rarity of the presentation of acute bilateral foot drop is underlined if we consider that between the authors, we have been in practice for a collective of >100 years.

## Conclusion

Bilateral foot drop can occur in chronic fashion as a sequela of systemic disease. However, when *bilateral* foot drop occurs *acutely*, we encourage the consideration of degenerative spinal disease in the differential diagnosis. Communicating this with colleagues in receiving/referring specialties might be prudent. Relevant discopathy was observed from L2/3 to L5/S1. The prognosis seems to be pretty good with respect to recovery of the foot drop, especially if partial at presentation and if treated within 48 h.
